# Comparison of Clinical Characteristics and Peripheral Blood Tests of COVID-19 and Influenza B Patients

**DOI:** 10.4269/ajtmh.22-0620

**Published:** 2023-03-13

**Authors:** Shan Yu

**Affiliations:** 1 Department of Infectious Diseases, Renji Hospital, Affiliated to Shanghai Jiaotong University School of Medicine, Shanghai, China;; 2 Shanghai University of Traditional Chinese Medicine, Shanghai, China

## Abstract

This study aimed to investigate the differences in clinical features and routine peripheral blood testing between coronavirus disease 2019 (COVID-19) infection and influenza B infection. Patients with COVID-19 and influenza B admitted to our fever clinic from January 1, 2022 to June 30, 2022 were recruited. A total of 607 patients were included (301 with COVID-19 infection and 306 with influenza B infection). The results of a statistical analysis showed that 1) patients with COVID-19 infection were older and had a lower temperature and shorter duration from fever onset to clinic visit than patients with influenza B infection; 2) apart from fever, viral infection symptoms appeared to be more common in patients with influenza B infection than in patients with COVID-19 infection, including sore throat, cough, muscle aches, weeping, headache, fatigue, and diarrhea (*P* < 0.001); and 3) compared with patients with influenza B infection, patients with COVID-19 infection had higher numbers of white blood cells and neutrophils but lower numbers of red blood cells and lymphocytes (*P* < 0.001). In summary, several important differences were identified between COVID-19 and influenza B, which may help to guide clinicians in their initial diagnosis of these two respiratory viral infections.

## INTRODUCTION

Since late 2019, severe acute respiratory syndrome coronavirus 2—officially named coronavirus disease 2019 (COVID-19)—has spread around the world, seriously endangering human health and becoming a public health epidemic worldwide.^1^^,^^2^ As the latest variant first detected in Botswana and South Africa, Omicron spread quickly across South Africa and worldwide, with at least 77 countries reporting Omicron cases by the end of 2021.^3^^,^^4^ The General Office of the National Health Commission in China updated guidelines for COVID-19 diagnosis and treatment in its ninth edition, giving people a better understanding of the disease. Once infected with COVID-19, patients usually have a fever, dry cough, and fatigue,^5^ which is similar to symptoms of influenza infection. Influenza is an acute respiratory infection caused by influenza viruses, mainly types A, B, and C.^6^^,^^7^ Influenza B, which is the primary influenza virus in China, has a strong pathogenicity and a high incidence in winter and spring.^8^^,^^9^

In early 2022, there was an outbreak of COVID-19 in Shanghai due to the Omicron variant. Of note, COVID-19 outbreak seasons coincide with the high-incidence seasons for influenza B. The timing of onset, symptoms, incubation period, and mode of transmission of COVID-19 are similar to those of influenza B, including fever as the main first symptom. However, the diagnosis of COVID-19 and influenza B is based mainly on viral nucleic acid testing, a lengthy and expensive method.^10^ Here, we performed a retrospective study among patients with COVID-19 or influenza B infection in the fever clinic to explore differences in their clinical characteristics and peripheral blood tests, providing practical assistance in clinical diagnosis and treatment.

## MATERIALS AND METHODS

### Patients and methods.

A retrospective study was performed in the fever clinic of Renji Hospital, which is affiliated with Shanghai Jiaotong University School of Medicine, between January 1, 2022 and June 30, 2022. Influenza B and COVID-19 were diagnosed by real-time reverse transcription polymerase chain reaction. The Institutional Ethics Board of the hospital approved the study. A trained group of medical care workers reviewed patients’ electronic medical records to extract data on demographics, medical history, clinical symptoms, and blood tests for hematology and biochemistry.

### Statistical analysis.

Categorical variables were expressed as frequency and percentages (%), and continuous variables were expressed as median (interquartile range, IQR). Mean values and percentages were compared among different groups by analysis of variance or χ^2^ tests. Categorical variables between groups were analyzed using the Mann–Whitney *U* test. All statistical analyses were performed using SPSS 24.0 (SPSS Inc., Chicago, IL). A *P* value < 0.05 was considered statistically significant.

## RESULTS

A total of 607 subjects were enrolled in the study, of whom 301 patients were afflicted with COVID-19 and 306 patients were afflicted with influenza B. Overall, 51.1% of subjects were male, and the median age was 42 years (IQR: 30–54 years). The median temperature was 38.0 °C (IQR: 37.4–38.6 °C), and the median duration from fever onset to clinic visit was 1.5 days (IQR: 1–2 days). Compared with subjects with influenza B infection, patients with COVID-19 infection were older (51 years versus 33 years) and had a lower temperature (37.7 °C versus 38.3 °C) and shorter duration from fever onset to clinic visit (1 [IQR: 1–1] day versus 1 [IQR: 1–2] day) (Table 1).

**Table 1:** Comparison of demographic features between COVID-19 and influenza B patients

Demographic characteristic	Total (*N* = 607)	COVID-19 (*N* = 301)	Influenza B (*N* = 306)	*P* value*
Male gender, *n* (%)	310 (51.1)	154 (51.2)	156 (51.0)	0.932
Age, years, median (IQR)	42 (30, 54)	51 (32, 66)	33 (29, 39)	< 0.001
Temperature (°C)	38.0 (37.4, 38.6)	37.7 (37, 38.5)	38.3 (37.8, 38.7)	< 0.001
Time from fever onset to clinic visit, days	1.5 (1, 2)	1 (1, 1)	1 (1, 2)	< 0.001

COVID-19 = coronavirus disease 2019; IQR = interquartile range.

* Comparison between COVID-19 and influenza B patients.

Clinical symptoms were also compared in COVID-19 and influenza B patients, including sore throat (45.9% versus 82.4%), cough (44.5% versus 78.4%), muscle aches (43.5% versus 68.3%), weeping (38.2% versus 53.6%), headache (35.9% versus 65.4%), fatigue (21.3% versus 73.5%), and diarrhea (2.0% versus 13.1%) (*P* < 0.001) (Table 2). These results indicate a significantly lower proportion of clinical symptoms in COVID-19 patients than in influenza B patients.

**Table 2:** Proportion of clinical symptoms in COVID-19 and influenza B patients

Clinical symptoms	COVID-19 (*N* = 301), *n* (%)	Influenza B (*N* = 306), *n* (%)	χ^2^	*P* value*
Sore throat	138 (45.9)	252 (82.4)	88.039	< 0.001
Cough	134 (44.5)	240 (78.4)	73.788	< 0.001
Muscle aches	131 (43.5)	209 (68.3)	37.810	< 0.001
Weeping	115 (38.2)	164 (53.6)	14.468	< 0.001
Headache	108 (35.9)	200 (65.4)	52.757	< 0.001
Fatigue	66 (21.9)	225 (73.5)	161.897	< 0.001
Diarrhea	6 (2.0)	40 (13.1)	26.590	< 0.001

COVID-19 = coronavirus disease 2019.

* Comparison between COVID-19 and influenza B patients.

Furthermore, the comparison disclosed that COVID-19 patients had higher numbers of white blood cells (5.90 versus 5.43) and neutrophils (4.07 versus 3.53) than influenza B patients, but lower numbers of red blood cells (4.59 versus 4.81) and lymphocytes (0.98 versus 1.13) (*P* < 0.001). However, the statistical analysis showed no significant difference in platelets, monocytes, and eosinophils between COVID-19 and influenza B patients (*P* > 0.05) (Table 3).

**Table 3:** Comparison of peripheral blood tests between COVID-19 and influenza B patients

Number	COVID-19 (median [IQR], 10^9^/L)	Influenza B (median [IQR], 10^9^/L)	*Z*	*P* value*
WBC	5.90 (4.82, 7.26)	5.43 (4.26, 6.565)	−3.836	< 0.001
RBC	4.59 (4.29, 4.89)	4.81 (4.42, 5.14)	−4.499	< 0.001
PLT	198 (166, 230)	197 (169, 229.5)	−0.388	0.698
NEUT	4.07 (3.13, 5.6)	3.53 (2.595, 4.54)	−5.076	< 0.001
LYM	0.98 (0.66, 1.38)	1.13 (0.87, 1.445)	−4.457	< 0.001
MON	0.53 (0.4, 0.71)	0.55 (0.43, 0.73)	−1.380	0.168
EOS	0.02 (0.01, 0.06)	0.03 (0.01, 0.06)	−1.760	0.078

COVID-19 = coronavirus disease 2019; EOS = eosinophils; LYM = lymphocytes; MON = monocytes; NEUT = neutrophils; IQR = interquartile range; PLT = platelets; RBC = red blood cells; WBC = white blood cells.

* Comparison between COVID-19 and influenza B patients.

## DISCUSSION

COVID-19 is still a widespread epidemic around the world. During the high-incidence influenza seasons, the prevention of the epidemic is even more challenging. Considering the resemblance between the two diseases, especially fever, fever clinics serve a vital role as the sentinel sites for respiratory infections.

In the present study, the prevalence of COVID-19 and influenza B infection was similar in sex, consistent with the finding of Yu et al.^11^ However, the age of COVID-19 patients was greater than that of influenza B patients. This may be due to the Omicron strain, which spreads faster than previous variants, and the fact that COVID-19 is prevalent in elderly and chronically ill patients.^12^ This finding also supports an earlier study that showed young and middle-aged people were susceptible to influenza B.^13^ In patients attending fever clinics, the temperature of those with influenza B is commonly higher than the moderate fever of patients with other general viral infections. In addition, our study included COVID-19 patients with a low to moderate fever. Nevertheless, the shorter time from fever onset to consultation observed in patients with COVID-19 versus those with influenza B may be mainly attributable to increased concern and anxiety about COVID-19 and its effects.^14^ This finding indicates that our technology and the speed of detection of novel coronavirus have progressed and that our diagnostic ability has gradually improved relative to the initial stages of the epidemic.^15^

Several studies showed that early clinical symptoms were atypical for both patients, accompanied with fever, cough, a runny nose, diarrhea, or muscle aches.^16^^,^^17^ In the current study, a comparison of these main symptoms revealed that sore throat and cough, the two most prevalent symptoms in influenza B patients, were substantially less prevalent in COVID-19 patients. These results also indirectly support the current definition of influenza-like illness established by the WHO guidelines, for which sore throat and dry cough are influenza patients’ main symptoms. Other studies also found that Omicron differs from other variants of novel coronaviruses, being less symptomatic and having a lower mortality rate.^18^ The low incidence of symptoms in patients with COVID-19 in this study may be due to clinic visits occurring in the early stages of the disease.

Blood cell analysis is one of the most commonly used primary screening tools in clinical practice, where leukocytes, neutrophils, and lymphocytes can promptly reflect disease pathology and immune status. In this study, more than 95% of patients in both groups had normal or low white blood cell counts on the day of clinic visit. The leukocyte and neutrophil counts were higher in patients with COVID-19 than in those with influenza B, whereas the lymphocyte count was lower. This result is consistent with the diagnostic reference standards for both diseases and is identical to the results of clinical studies.^19^^,^^20^ In addition, we found red blood cells plummeted in COVID-19 patients. Early cases of COVID-19 also showed reduced bone marrow hematopoietic cell counts.

## STUDY STRENGTHS AND LIMITATIONS

The sample size in this study was small, and the laboratory parameters were incomplete. In addition, the study did not allow for dynamic detection of changes in peripheral blood. Detailed and specialized blood tests for the early diagnosis and assessment of COVID-19 are also lacking. Therefore, more studies designed to validate these findings are warranted.

## CONCLUSION

We suggest the use of COVID-19 testing in patients with a low to moderate fever and decreased red blood cell and lymphocyte counts, but influenza B testing in patients with a moderate to high fever and a sore throat, cough, and decreased white blood cell and neutrophil counts. Considering that virus antigen testing methods are unavailable in primary care hospitals, the criteria may help differentiate these two diseases and expedite timely referral to a higher-level hospital for relevant pathogenic testing. Nevertheless, given the overlap in clinical and laboratory manifestations, testing for both infections should be done whenever feasible and when both viruses are circulating.
